# How urban proximity shapes agricultural pest dynamics: a review

**DOI:** 10.1002/ps.8671

**Published:** 2025-01-20

**Authors:** Lior Blank

**Affiliations:** ^1^ Department of Plant Pathology and Weed Research, Agricultural Research Organization Volcani Institute Rishon LeZion Israel

**Keywords:** citizen science, community gardens, landscape ecology, pest management, source–sink dynamics, spillover, urban agriculture, urban heat island, urban–rural gradients

## Abstract

Agricultural landscapes adjacent to human settlements are subject to unique ecological dynamics that influence pest populations, yet the complexity of these relationships remains relatively underexplored. This review synthesizes current knowledge on the impacts of urban proximity on agricultural plant pathogen pest dynamics, focusing on spatial distribution patterns, theoretical frameworks from landscape ecology, and the specific mechanisms driving these interactions. The urban heat island effect, habitat fragmentation, and human activities contribute to altered microclimates, reduced natural predator populations, and increased pest proliferation near settlements. Additionally, regulatory constraints on pest control near human communities further complicate management efforts. The role of urban environments as potential sources of agricultural pests is analyzed through empirical case studies, highlighting both predictable patterns and varying outcomes depending on specific local conditions. Gaps in understanding the movement of pests across urban–agricultural boundaries are discussed, alongside recommendations for future research aimed at enhancing pest control strategies in these complex landscapes. © 2025 The Author(s). *Pest Management Science* published by John Wiley & Sons Ltd on behalf of Society of Chemical Industry.

## Graphical abstract

This review explores how proximity to human settlements affects agricultural pest and pathogen dynamics, highlighting the roles of microclimatic conditions, biological aspects, and management effects. It underscores the need for integrated pest management strategies to address these complex interactions and enhance sustainable agriculture.

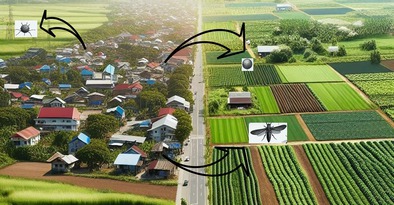

## 1 INTRODUCTION|

Agriculture forms the backbone of global food security, yet it faces persistent challenges from pests (specifically arthropods and plant pathogens in this article) that threaten crop yields and quality.^1^ Weeds were not included due to their distinct ecological and management characteristics, which require separate consideration. As human populations continue to expand and agricultural landscapes become increasingly intertwined with human settlements, understanding the ecological dynamics at this interface becomes important for sustainable agriculture. The spatial arrangement of agricultural fields in relation to human settlements has long been recognized as a factor influencing crop health and productivity^2^, ^3^ and the complexities of these relationships and their implications for pest management have been studied systematically in the last two decades.^4^

## 2 THEORETICAL FRAMEWORK

### 2.1 Landscape ecology principles

Landscape ecology provides a framework for studying the spatial dynamics of agricultural pests. This discipline emphasizes the importance of spatial heterogeneity and the interactions between different landscape elements in shaping ecological processes.^2^, ^3^, ^5^ In agricultural contexts, the landscape can be conceptualized as a mosaic of different land‐use types, including crop fields, orchards, natural or semi‐natural habitats, and human settlements. The composition, configuration, and connectivity of these landscape elements can influence the distribution and abundance of both pest species^4^ by providing a range of habitats and resources, enhancing connectivity for dispersal, and creating microhabitats that support survival and reproduction. The diversity and arrangement of different habitat types within a landscape, such as crops, natural vegetation, and non‐crop areas (e.g., settlements, forests, or grassland), create varying conditions that can either promote or inhibit pest distribution.^6^, ^7^

Landscape heterogeneity can increase pest abundance by providing a variety of habitats and resources. For example, pests that thrive in monoculture fields may benefit from nearby semi‐natural habitats that offer alternative food sources or overwintering sites, during unfavorable periods when crops are unsuitable or when conditions are unfavorable for spread of the pest. These adjacent habitats can help pests survive and later recolonize crop fields quickly when conditions improve.^8^ The presence of diverse habitats can also facilitate the movement of pests across the landscape, increasing their potential to infest multiple fields and spread diseases.^9^, ^10^, ^11^ This connectivity can enhance the dispersal of pathogens between infected and healthy plants, leading to a broader distribution of plant diseases,^3^ or the movement of pests between adjacent orchard types in relation to host phenology.^12^

Additionally, the composition of the landscape suggests that the amount and type of crop and non‐crop habitats can influence pest dynamics by affecting host availability persistence. Landscapes with large areas of suitable host plants or crops that are susceptible to specific pests can support higher pest populations and increase the risk of pest outbreaks. For instance, fields surrounded by non‐crop habitats that are alternative hosts for pests or diseases can serve as reservoirs, maintaining high levels of pest pressure on adjacent crops.^13^

Landscape configuration, which refers to the spatial arrangement of different habitat patches, also plays a critical role in pest distribution. For example, landscapes with fragmented patches of semi‐natural habitats may limit the movement of natural enemies, thereby reducing their ability to control pest populations effectively.^14^ In contrast, pests that can utilize multiple habitats or require resources from different land‐cover types may benefit from a landscape that provides easy access to diverse habitats.^3^ The connectivity of habitat patches is particularly important for species that require continuous habitats for movement; well‐connected landscapes can facilitate the spread of both pests across large areas.^10^

### 2.2 Source–sink dynamics and metapopulation theory

Studying agricultural pests in the realm of ecological theory is important for generalizing patterns and processes.^15^ The concepts of source–sink dynamics and metapopulation theory are particularly relevant to understanding pest dynamics in agricultural landscapes.^16^ Source habitats are areas where local reproduction exceeds mortality, while sink habitats are areas where mortality exceeds local reproduction. In the context of pest ecology, human settlements or nearby natural habitats may act as sources, continuously supplying pests to nearby agricultural fields.

Metapopulation theory extends this concept to networks of habitat patches, describing how local extinctions and recolonizations can lead to the overall persistence of a species at the landscape scale.^17^ This framework is particularly useful for understanding the dynamics of pests that may persist in the landscape even when individual fields are cleared or rotated.

## 3 SPATIAL DISTRIBUTION OF PESTS

The spatial distribution of pests in agricultural ecosystems is inherently complex, spanning multiple scales from individual fields to entire landscapes. This complexity arises from the interplay of various biotic and abiotic factors that shape the environment in which these organisms exist and proliferate. At the landscape level, regional climate patterns,^9^, ^18^, ^19^, ^20^ the mosaic of different crop types and rotations,^12^ and the presence of natural or semi‐natural habitats^21^, ^22^, ^23^, ^24^, ^25^ all contribute to the heterogeneity that influences pest distributions.

Within this broader context, local‐scale variations further modulate pest populations. Field‐specific factors such as soil characteristics, microclimatic conditions, and topography create unique niches that can either promote or inhibit the establishment and spread of these organisms,^22^, ^24^ and uneven distribution within the field is also prevalent,^26^, ^27^ and is true also for weeds.^28^, ^29^, ^30^, ^31^ Moreover, human actions, including farming practices and pest management approaches, add another layer of complexity to this spatial mosaic.^32^, ^33^

Understanding these multi‐scale spatial dynamics is crucial for developing effective pest management protocols. The distribution patterns observed at different scales – from individual fields to entire agricultural regions – reflect the cumulative effects of ecological processes, environmental conditions, and human actions.^22^ This spatial heterogeneity not only affects the current distribution of pests but also influences their potential spread and the efficacy of control measures.^34^, ^35^ By examining these spatial patterns and the factors that drive them, researchers and farmers can gain insights into the ecological mechanisms underlying pest dynamics. This knowledge is essential for predicting outbreaks, optimizing monitoring efforts, and designing the agricultural landscape.

This review aims to synthesize current knowledge on how the proximity of fields to human settlements affects the incidence, severity, and management of agricultural pests. It examines theoretical frameworks, empirical evidence, and underlying mechanisms to provide a comprehensive overview of this important aspect of agricultural landscape ecology.

### 3.1 Urban ecosystems as source of agronomically important pests

Urban ecological studies typically focus on the patterns of abundance and diversity of species within cities.^36^, ^37^ However, the mechanisms governing the interplay between urban environments and the surrounding landscape received little attention. Urban gardens and recreation areas vary greatly from traditional crop fields and orchards in both climatic conditions, vegetation management and pest dynamics.^38^, ^39^

Urban environments are intricate ecosystems that can support pests of agronomical significance. These settings offer various niches conducive to the life cycles of organisms affecting plant health. Internal sources of such pests within urban areas include urban agriculture,^40^ where rooftop farms, vertical gardens, and other urban cultivation forms can harbor pests. Green roofs, while environmentally beneficial,^37^, ^41^ can also be a source of pests. Urban parks can also serve as sources of agronomically important pests, with their diverse array of plant species, including native and ornamental plants, which can support a wide range of pests. Private gardens and backyards, common in urban areas, can be significant sources of pests, as homeowners may inadvertently introduce pests through infested plants or contaminated soil.^42^ Another potential source are plant nurseries, which are critical entry points for pests into urban areas, as the high turnover of plant material and the movement of plants between nurseries and customers can spread harmful pests. In addition, community gardens, shared spaces where individuals grow food and ornamental plants, can be hotspots for detrimental organisms due to the variety of plants grown and the varying levels of gardening expertise among participants. Shared tools and close plant spacing can contribute to the spread of diseases. Understanding these internal sources is essential for developing effective integrated pest management strategies tailored to urban environments.

### 3.2 Mechanisms of urban proximity driving pest dynamics

#### 3.2.1 Abiotic drivers

Studies have shown that insect densities can be higher in urban green spaces, with greater canopy cover.^43^ However, urbanization radically alters land surfaces, habitat structure and ecological function well beyond the bounds of the city.^44^

##### 3.2.1.1 Urban heat island

Human settlements significantly modify natural landscapes, introducing novel habitats, altering resource availability, and changing local climate conditions (Table 1). These modifications have far‐reaching effects on local and regional biodiversity, including agricultural pests.^3^ The concept of ‘urban–rural gradients’ has been particularly useful in understanding how human‐modified landscapes affect ecological processes,^45^ recognizing that the intensity of human influence typically decreases with distance from urban centers. The urban heat island (UHI) effect, a well‐known consequence of urbanization,^46^, ^47^ can alter temperature regimes in cities, potentially impacting disease prevalence and severity.^48^, ^49^ For instance, UHI has been linked to increased severity of powdery mildew on English oak in Europe.^50^ Greater temperature variability in urban environments, including the UHI effect,^46^ is linked to higher plant damage, particularly from diseases, demonstrating the critical role of local microclimate in disease dynamics.^48^ Urban areas typically experience higher temperatures – 1–12 °C warmer than surrounding rural areas^47^ – and greater interday variation, leading to increased damage from diseases like powdery mildew, and Brassica pathogens. These effects are driven by the biology of plant pathogens, with fungal and oomycete diseases relying on temperature and moisture for spore dispersal.^10^, ^51^ For example, powdery mildew thrives in warm, dry conditions,^52^ while several Brassica diseases spread through air moisture and depend on soil temperature and humidity.^53^ Additionally, urban forests show that even under intensive management, trees remain vulnerable to pests during heat waves.^54^ In addition, the UHI effect can extend the growing seasons, potentially allowing pests to complete more generations in a single season and thereby increasing their population density.^55^

**Table 1:** Summarizing the urban factors influencing agricultural pest dynamics

Urban factor	Impact on pests	Examples
*Abiotic drivers*		
Urban heat island effect	Increased temperature and extended growing season allow pests to complete more generations	Powdery mildew on English oak^50^
Temperature variability	Higher plant damage	Powdery mildew^52^ and Brassica pathogens^53^
Air quality (pollution)	Increase plant susceptibility to various pathogens due to stress responses; degradation of plant cuticle	*Botrytis cinerea* ^58^ and *Alternaria brassicicola* ^59^
Light pollution	Disrupts insect growth, distribution, foraging, mating, and predation	Pea aphid^71^
Noise pollution	Disrupts insect communication, mating, and predator–prey interactions	Lady beetles^76^
*Biotic drivers*		
Plant susceptibility and pest development	Alters plant disease dynamics and pathogen–host interactions	Mildew epidemics^82^
Reduction of natural predators	Disruption ecosystem balance	Reduction in populations of ladybugs, spiders, and predatory beetles^84^
*Management drivers*		
Limitations of aerial spraying	Reliance on less efficient ground‐based methods for pest control	Mediterranean fruit fly^22^
Human activities in settlements	Facilitation of pest introductions (through transportation, trade, and agriculture)	Citrus greening in south Florida^93^; late blight outbreaks in north‐eastern United States^42^
Lack of awareness and monitoring	Undetected infestations	Undetected infestations spreading beyond urban boundaries^85^, ^89^
Untreated fruit trees in urban areas	Serve as reservoirs for pests	Mediterranean fruit fly^22^ and Mal secco^24^

While elevated temperatures can initially favor some insect species by extending growing seasons and boosting metabolic rates, exceeding thermal optima for prolonged periods poses significant threats to insect abundance, occurrence, and potential for damage. Extreme temperatures may push some pests beyond their physiological limits, leading to a cascade of negative consequences. Higher temperatures can increase metabolic costs disproportionately, leading to thermal injuries, developmental failures, reduced fecundity, and ultimately decreased fitness and mortality.^56^ Extreme heat can also impair dispersal capacity, hindering escape from unfavorable conditions.^56^ The cumulative impact of these factors can result in population crashes for many insect species. This underscores the complex and often detrimental effects of UHIs.

##### 3.2.1.2 The impact of urban air quality on plant susceptibility to diseases

Air quality in urban areas significantly impacts not only human health but also plant susceptibility to pathogens. Throughout the past century, extensive research has documented plant injury and damage due to air pollution.^57^ The impact of various air pollutants, such as ozone, sulfur dioxide, acid rain, and particulates, on plant pathogens has been studied and documented in the literature. For example, Leone and Tonneijck^58^ found that ozone induces the predisposition of bean leaves to *Botrytis cinerea*. Khan and Khan^59^ found that Indian mustard (*Brassica juncea*) became more susceptible to *Alternaria brassicicola* following exposure to high concentrations of sulfur dioxide.

Urban air pollution can cause various stress responses in plants. For instance, pollution‐induced stress can stimulate the production of small organic plant molecules such as ethane and ethylene.^57^ Moreover, studies have observed surface wax degradation in plants following exposure to exhaust gases, attributed to organic hydrocarbons and nitrogen oxides (NO*x*),^60^ or more specifically, to lipophilic aromatic hydrocarbons associated with vehicle emissions.^61^ This degradation is particularly concerning because the plant cuticle serves several crucial functions, including preventing excessive water loss, regulating solute uptake, protecting sensitive underlying tissues, and acting as a barrier to pathogens.^62^ However, plant cuticle is not just a physical barrier but also plays an active role in plant defense. It is involved in signaling pathways for growth and development and functions in the first layer of plant defense activating local and systemic acquired resistance against diverse pathogens.^63^ Consequently, the deterioration of this protective layer due to poor air quality in urban environments may increase plants' susceptibility to pathogen infections, potentially leading to more frequent and severe disease outbreaks in urban vegetation.

##### 3.2.1.3 Light pollution

Light pollution, particularly artificial light at night (ALAN), disrupts insect growth,^64^ alters distribution,^65^ reduces foraging efficiency,^66^ delays mating,^67^ increases predation risk,^68^ and affects hormone production,^69^ leading to population declines. However, ALAN can have several positive effects on insects, although these are often context‐dependent and may not outweigh the negative impacts on insect populations. ALAN can interfere with the behavior of nocturnal predators, such as bats that help control insect populations.^70^ Additionally, monochromatic red illumination has been observed to reduce the frequency at which parasitoid wasps locate their pea aphid hosts.^71^ It can also alter behavior patterns, potentially aiding in mate finding by bringing individuals together in illuminated areas, which could lead to increased encounters under certain conditions.

##### 3.2.1.4 Noise pollution

Anthropogenic sound is increasingly acknowledged as a significant factor contributing to global environmental change, affecting both urban and rural ecosystems.^72^ The study by Morley *et al*.^73^ highlights the significant impact of anthropogenic noise on invertebrates, particularly insects. Insects have evolved diverse auditory structures, allowing them to detect sound across a wide range of frequencies. Many insects use sound for communication, mate attraction, and predator avoidance, making them susceptible to noise interference.^74^ Moreover, noise can disrupt acoustic communication, which is crucial for mate location and courtship in many insect species. However, noise pollution from traffic and industrial activities can disturb predator species, reducing their ability to hunt effectively,^75^ positively affecting some insects. For example, lady beetles exposed to noise pollution were less effective predators, leading to higher aphid density and reduced plant biomass.^76^

In addition to the impact of anthropogenic sound on arthropod communication, it is important to consider substrate‐mediated communication, which involves seismic vibrations. Urban environments not only produce sounds but also generate seismic vibrations through various activities such as construction, traffic, and industrial operations.^77^ The vibratory sensory channel is important for arthropods, that use vibrations for conspecific communication,^78^ prey detection,^79^ and predator evasion.^80^

#### 3.2.2 Biotic drivers

##### 3.2.2.1 Plant susceptibility and pest development

Increased human activities in urban areas, such as mowing, can directly impact plant health and indirectly affect disease susceptibility.^81^ A recent study found that mildew epidemics on *Plantago rugelii* started earlier and achieved greater prevalence in more urban sites, highlighting the complex interactions between urbanization and plant disease dynamics.^82^ While the effects of urbanization on plant fitness are still not fully understood, emerging evidence suggests that urban environments can significantly alter pathogen–host interactions through various ecological and evolutionary mechanisms.^83^ Although this article does not specifically address agronomic pests, it provides valuable insights into how urbanization influences pathogen/pest–host interactions. Urban environments can increase genetic drift and restrict gene flow, leading to reduced genetic diversity in host populations. Additionally, elevated mutation rates due to pollution may accelerate the evolution of both pathogens and hosts. The article also highlights that divergent selection between urban and non‐urban environments can drive adaptive evolution, potentially altering host susceptibility and resistance to pathogens.

##### 3.2.2.2 Reduced natural predators

Human activities in settlements can severely reduce the populations of natural predators and disrupt ecosystem balance, leading to weakened pest control and increased pest abundance in nearby areas. Urbanization fragments natural habitats by replacing forests, wetlands, and open spaces with roads, buildings, and other infrastructure, in addition to the effects of noise and light pollution, already mentioned, limiting the spaces where predators such as birds, bats, and beneficial insects can thrive. For instance, predators like ladybugs, spiders, and predatory beetles rely on green spaces and diverse ecosystems to control pests, but in densely populated areas, their habitats are often destroyed or reduced.^84^

By reducing the abundance of natural predators, human activities in settlements create an environment where pests can multiply unchecked. This imbalance results in higher occurrences of pests in agricultural fields and natural areas adjacent to settlements, as natural pest control mechanisms are weakened or lost entirely.

While urban conditions can reduce natural predator populations due to habitat fragmentation and pollution, they can also facilitate the growth and abundance of natural predators. The UHI effect, for example, can extend the active periods of both pests and their natural enemies, potentially increasing predator populations.^55^ Additionally, the lack of intensive pest management in urban areas can allow natural predator populations to thrive, as they are not subjected to frequent pesticide applications.^85^ In addition, urban green spaces and gardens can serve as refuges for these predators, supporting their populations and enhancing their role in pest control.^40^

#### 3.2.3 Management drivers

##### 3.2.3.1 Human activities driving pest proliferation in settlements

A study on *Ceratocystis platani*, the causal agent of canker stain in plane trees, found higher inoculum concentrations in urban environments compared to rural areas.^86^ The researchers hypothesized that intensive tree sanitation practices in cities, such as pruning and felling, resulted in increased airborne inoculum dispersed through the abundant sawdust produced during these activities. Krasnov *et al*.^23^ showed inconsistent relationships between proximity to urban communities and pest occurrence. For the grape powdery mildew (*Uncinula necator*) proximity to urban areas had varied effects over different years, indicating that urban proximity might sometimes enhance and other times reduce pest levels. This inconsistency suggests that other unmeasured factors might be interacting with urban proximity to influence pest dynamics. These findings illustrate the complex and often inconsistent impact of proximity to human settlements on agricultural pest dynamics. While some studies show a clear positive correlation, others reveal inconsistent or weak associations, indicating the influence of additional, unmeasured factors.

In addition, urban infrastructure like road networks may facilitate pathogen dispersal, as evidenced by higher fungal infection rates in *Plantago lanceolata* populations along roadsides^87^ and an adult *Philaenus spumarius*, the primary vector of *Xylella fastidiosa* subsp. *pauca* was found alive after adhering to a moving car for over 40 km.^88^

##### 3.2.3.2 The lack of effective pest control in urban areas

The absence of awareness and limited resources for monitoring and controlling plant pests in urban settlements, unlike in agricultural regions, creates an environment where infestations can go undetected and unchecked.^85^ In agricultural settings, extensive resources are dedicated to pest management, including regular monitoring, integrated pest management strategies, and access to pesticides and biological control methods. However, urban settlements often lack these structured surveillance and management systems. Without early detection and rapid control measures, such infestations can eventually extend beyond urban boundaries, contributing to regional agricultural pest problems and necessitating costly containment and management efforts. In this context, citizen science may play a critical role in addressing the spread of plant pests.^89^

##### 3.2.3.3 Limitations of aerial spraying close to human communities

Regulations on aerial crop spraying near settlements are designed to protect human health, the environment, and non‐target species from the risks associated with pesticide use. These regulations vary by country and local jurisdiction but generally include key elements such as buffer zones aiming at preventing pesticide drift into sensitive areas. For example, New South Wales regulations require a 150‐m buffer zone around any public area.^90^ The state of New York has prohibited the aerial spraying of certain pesticides throughout its jurisdiction,^91^ a practice that was also banned by the European Union in 2009.^92^

## 4 EFFECTS OF URBAN PROXIMITY ON PESTS PROXIMITY ON PESTS IN RURAL AREAS: CASE STUDIES

### 4.1 Urban areas as sources of pests: case studies

#### 4.1.1 Diaphorina citri

Cross‐habitat spillover, where invasive species move between agroecosystems and natural habitats, was previously studied.^94^ Most research highlights asymmetric insect movement between agricultural areas and adjacent natural habitats.^95^ However, less is known about how common spillover of pests is from urban to agricultural ecosystems, or the processes governing it across space and time. Urban areas play a significant role in driving biological invasions in surrounding agricultural landscapes. For example, during the early phase of *Diaphorina citri* invasion, the Asian citrus psyllid, citrus groves near urban areas were more rapidly affected, and proximity to urban areas contributed positively to cumulative *D. citri* detections.^96^

#### 4.1.2 Phytophthora infestans

The 2009 late blight pandemic in the north‐eastern United States was characterized by an unusual synchronous onset in mid to late June.^42^ The introduction pathway was identified as infected tomato transplants distributed to garden centers in large retail stores across the region. By late June, it was evident that these infected transplants were being sold from Pennsylvania to Maine. The majority of garden center employees and home gardeners failed to recognize the symptoms of late blight, resulting in the planting of infected transplants. Subsequent reports confirmed the presence of late blight on commercial potatoes on Long Island, NY, and infected tomato transplants in numerous big box stores. It was determined that transplants from a single national supplier were infected.

#### 4.1.3 Phoma tracheiphila

Some studies have found more complex patterns. Ben‐Hamo *et al*.^24^ found a weak but significant positive association between the total area of urban terrain within 500 m of each orchard and the severity of Mal secco disease. This suggests that proximity to urban areas may contribute to higher disease rates in nearby orchards. The role of urban citrus trees as potential reservoirs for *Phoma tracheiphila*, the causal agent of Mal secco disease, remains a subject of debate. While untreated trees could theoretically serve as infection sources, this hypothesis is challenged by existing evidence. Studies have shown that *Phoma tracheiphilus* has a limited dispersal range, typically not exceeding a few dozen meters.^97^ This restricted spread capability suggests that urban trees may not significantly contribute to disease transmission in commercial orchards.

#### 4.1.4 Mediterranean fruit fly

Studies have reported higher pest abundance near settlements. For example, Krasnov *et al*.^22^ found that proximity to urban settlements was positively correlated with higher populations of the Mediterranean fruit fly in citrus orchards. The authors suggested that untreated fruit trees in urban areas might serve as reservoirs for pests, thus increasing pest pressure on nearby agricultural fields.

### 4.2 Disruption of applying control measures

#### 4.2.1 Mediterranean fruit fly

Krasnov *et al*.^22^ found that plots within human communities had higher Mediterranean fruit fly trap density (FTD) values, decreasing with distance and stabilizing around 250 m from the settlement boundary. The authors also found that aerial spraying significantly reduced FTD compared to ground‐based methods. However, due to potential health and environmental risks, aerial spraying is restricted near human settlements.^22^ This necessitates ground spraying up to 80 m from communities, where pest density is highest. As a result, the most effective control method cannot be used where it is most needed, relying instead on less efficient ground‐based methods.

### 4.3 Introducing of new pests

Human activities in settlements frequently facilitate pest introductions, as transportation, trade, and agriculture move potentially infested goods and materials. Villages and residential areas, serving as local agricultural and commercial hubs, often become sites for pest introductions, where suitable conditions enable these species to thrive and spread. Here are two examples.

#### 4.3.1 Citrus greening

In the United States, the first case of citrus greening was identified in residential areas of south Florida^98^ and the disease quickly spread across the state. Recently, both the disease and its vector, the Asian citrus psyllid (ACP), were detected in residential habitats along Florida's north‐western Gulf Coast. This rapid spread of ACP has been partly attributed to the mix of expansive commercial citrus groves with largely unmanaged backyard citrus trees. This unmanaged residential citrus creates an ideal environment for ACP transmission.^93^, ^99^

#### 4.3.2 Xylella fastidiosa

Historically, *Xylella fastidiosa*‐induced diseases in economically significant crops were restricted to the American continent. This situation has evolved with the emergence of various subspecies of the pathogen in Europe. Notably, in 2015, four *Polygala myrtifolia* samples in France, were found to be infected with *X. fastidiosa* subsp. *pauca*, in a botanical garden.^100^ In October 2016 *X. fastidiosa* subsp. *fastidiosa* was found on three young cherry trees in a garden in the center of Mallorca, where it is considered now established.^101^

## 5 POTENTIAL ACTIONS FOR MANAGING URBAN PESTS

### 5.1 In urban areas

Urban areas present unique challenges and opportunities for managing pests. Effective management requires a multifaceted approach that includes monitoring, sanitation, public education, and centralized management. Here are some potential actions that can be taken.

Monitoring and citizen science – regular monitoring is essential for early detection and management of pests. This can be achieved through systematic surveys, trapping, and data collection.

Fruit sanitation – proper fruit sanitation can significantly reduce the habitats and food sources for pests and include removing fallen and overripe fruits (to eliminate potential breeding sites for pests), encouraging timely harvesting of fruits, and proper disposal of infested or diseased fruits.

Public education – informing the public about the risks and how to identify pests, and encouraging community involvement, can aid in management efforts.

Centralized management – a coordinated effort by government or city councils can enhance the effectiveness of pest management and include policy development, resource allocation, and intervention.

By implementing these actions, urban areas can reduce the occurrence and abundance of pests, leading to healthier environments and communities. It is important to note that these measures should be part of an integrated pest management strategy that emphasizes sustainable and environmentally friendly practices.

### 5.2 In adjacent fields

Farmers should consider implementing extra pest monitoring and control measures along the edges of fields adjacent to urban areas. Additionally, strategic decisions about crop placement, by selecting crops that are less susceptible to urban‐associated pests, can also be beneficial to mitigate the impact of urban land use, as the negative effects of urban environments on pests often diminish with distance.^22^

## 6 CONCLUSIONS

Despite substantial progress in understanding the interactions between urban and agricultural ecosystems, critical gaps remain. Future research should prioritize investigating the processes that drive the movement of pests across urban‐agricultural boundaries. Some key knowledge gaps include: What is the maximum distance at which urban areas exert effects on agricultural fields concerning pest spread? How can pest populations be effectively controlled within urban environments to prevent them from impacting nearby agricultural areas? Are there specific crops that are better suited to being planted near settlements to minimize the impact of urban areas as sources of pests? Do urban areas generally serve as pest sources across different climates and ecosystems?

Understanding the interactions between urban and agricultural environments is crucial for effective pest control. Monitoring urban areas near crop fields throughout the growing season, for example, may help reduce risks, and placing traps for flies and other pests in gardens and recreational areas could prove beneficial. Communities should also be educated about the risks posed by untreated gardens and be encouraged to adopt practices like fruit sanitation. Guidance on which fruit trees to plant, as well as identifying plants that can serve as hosts to pests, can further minimize the risk of spreading diseases.

This review aims to elucidate how urban ecosystems can promote and facilitate pest population growth and their subsequent spread to adjacent agricultural fields. While it is acknowledged that urban conditions such as noise, pollution, and heat can also negatively impact and suppress pest populations, the primary focus is on the potential advantages pests may derive from these environments. This approach is intended to underscore the risks and challenges posed by urban proximity to agricultural pest management.

Managing the interface between urban and agricultural landscapes requires both proactive measures and community involvement. Landscape‐scale management is essential for addressing the public good challenges inherent in pest control, as coordinated efforts across multiple fields and stakeholders can lead to more effective strategies.^102^ By integrating monitoring, education, and research, it is possible to create more effective pest management strategies. This approach will contribute to healthier environments for both crops and communities, addressing the challenges posed by the interplay between urban and agricultural settings.

## Data Availability

Data sharing not applicable to this article as no datasets were generated or analysed during the current study.
